# Left renal vein graft and in situ hepatic perfusion in hepatectomy for complete tumor invasion of hepatic veins: hemodynamic optimization and surgical technique

**DOI:** 10.1007/s00423-022-02451-6

**Published:** 2022-01-31

**Authors:** Víctor Lopez-Lopez, Jose Garcia-Lopez, Dilmurodjon Eshmuminov, Roberto Brusadin, Asunción Lopez-Conesa, Luis Martinez-Insfran, Pedro Fernández-Fernández, Ricardo Robles-Campos

**Affiliations:** 1 Department of Surgery, HBP Unit, Virgen de la Arrixaca University Hospital, IMIB-Arrixaca, Murcia, Spain; 2 Department of Anesthesiology, Virgen de la Arrixaca University Hospital, IMIB, Murcia, Spain; 3 Department of Surgery and Transplantation, Swiss Hepato-Pancreato-Biliary (HPB) Center, University Hospital Zurich, Zurich, Switzerland; 4 Department of Surgery, Reina Sofía University Hospital, Murcia, Spain

**Keywords:** Vascular graft, Liver metastases, Extreme liver surgery, In situ hepatic perfusion

## Abstract

**Purpose:**

Assessing hepatic vein reconstruction using a left renal vein graft and in situ hypothermic liver perfusion in an extended liver resection.

**Methods:**

Patients included in this study were those with liver tumors undergoing curative surgery with resection and reconstruction of hepatic veins. Hepatic vein was reconstructed using a left renal vein graft. We describe the technical aspects of liver resection and vascular reconstruction, the key aspects of hemodynamic management, and the use of in situ hypothermic liver preservations during liver transection (prior to and during vascular clamping).

**Results:**

The right hepatic vein was reconstructed with a median left renal venal graft length of 4.5 cm (IQR, 3.1–5.2). Creatinine levels remained within normal limits in the immediate postoperative phase and during follow-up. Median blood loss was 500 ml (IQR, 300–1500) and in situ perfusion with cold ischemia was 67 min (IQR, 60.5–77.5). The grafts remained patent during the follow-up with no signs of thrombosis. No major postoperative complications were observed.

**Conclusion:**

Left renal vein graft for the reconstruction of a hepatic vein and in situ hypothermic liver perfusion are feasible during extended liver resection.

**Supplementary Information:**

The online version contains supplementary material available at 10.1007/s00423-022-02451-6.

## Introduction

The treatment of choice for most primary and metastatic liver tumors is liver resection [1, 2]. A successful liver surgery is dependent on sufficient liver mass, adequate arterial perfusion, and venous outflow in addition to intact biliary drainage. Depending on the tumor location, a hepatic vein resection might be necessary for a radical surgery. In the past, liver tumors with involvement of hepatic veins were considered unresectable with poor prognosis. Recent improvements in surgical technique together with a greater understanding of the segmental liver anatomy, liver machine preservation, and perioperative patient management are making hepatic resection with involvement of hepatic veins a possibility.

Hepatic vascular exclusion and hepatic vein reconstruction are required during curative surgery for tumors invading hepatic veins [3–5]. Hepatic vascular exclusion can be total including the portal pedicle, the infra- and suprahepatic inferior vena cava (IVC) or partial, either by occluding the infrahepatic IVC and preserving hepatic flow or by occluding the portal pedicle and hepatic veins by leaving the IVC open. During hepatic vascular exclusion, the future liver remnant can be perfused to reduce ischemia and consequent injury during reperfusion. These measures are applied to minimize the need for blood transfusions, to minimize hepatic ischemia to assist in the hemodynamic stability of the patient. After hepatic vascular exclusion, the hepatic vein is resected and reconstructed. A variety of options have been proposed to replace veins in abdominal surgery [6–12]. Among them, autologous vein grafts are widely used due to their relatively common availability, low thrombotic rate, and low rate of infection. The left renal vein (LRV) graft is also a valuable option for the reconstruction of the hepatic veins during liver surgery [13–19]. The experience with regard to this is still lacking and a report on the topic will assist in understanding such a surgical technique.

This study reports the results of the hepatic vein resection reconstruction. Furthermore, the technical aspects of hepatic vascular exclusion, in situ liver perfusion to minimize liver ischemia, intraoperative hemodynamic monitoring, and hepatic vein reconstruction with autologous renal vein interposition will be described.

## Methods

### Patient selection

This is a retrospective study on a prospective database of liver resections performed from July 1985 to December 2020 at Virgen de la Arrixaca University Hospital, Murcia. Patients undergoing a resection of hepatic vein(s) with hepatic vascular exclusion were selected. Included patients were selected rigorously using the American Society of Anesthesiologist (ASA) score and had adequate cardiac, renal, and hepatic functions and were discussed in a multidisciplinary board. Computed tomography and magnetic resonance imaging were performed routinely to assess the tumor relation to hepatic veins with vena cava and collateral renal veins distribution.

#### Anesthetic management

- Hemodynamic management during liver transection. Restrictive fluid therapy was administered at 2 ml/kg/h of crystalloid solution (Plasmalyte®, Baxter Healthcare Ltd., UK) while maintaining cardiac index (CI) > 2.5 l/min/m^2^. In case of bleeding, albumin replacement (Albutein® 5%, Instituto Grifols, S.A. Barcelona, Spain) was provided at a 1:1 ratio.
- Hemodynamic management prior to vascular clamping: A supramaximal optimization protocol was conducted, consisting of the following: replacement fluid therapy with 1,000–1,500 ml of crystalloid solution until a systolic volume variation (SVV) < 10% is reached; dobutamine 2–5 mcg/kg/min for a CI > 4 l/min/m^2^; and norepinephrine 0.10–0.50 mcg/kg/min for a systemic vascular resistance index (SVRI) > 3,500 dynes-sec-m^2^/cm^5^.
- Management during vascular clamping. Portal vein clamping test was performed for 5 min. If the CI fell below 2.5 l/min/m^2^, the dosages of dobutamine and norepinephrine were increased to a maximum of 10 mcg/kg/min and 1 mcg/kg/min, respectively. IC > 2.5 l/min/m^2^ after 5 min of testing was considered favorable and the vascular resection phase was initiated.

#### Surgical technique

**Fig. 1:**
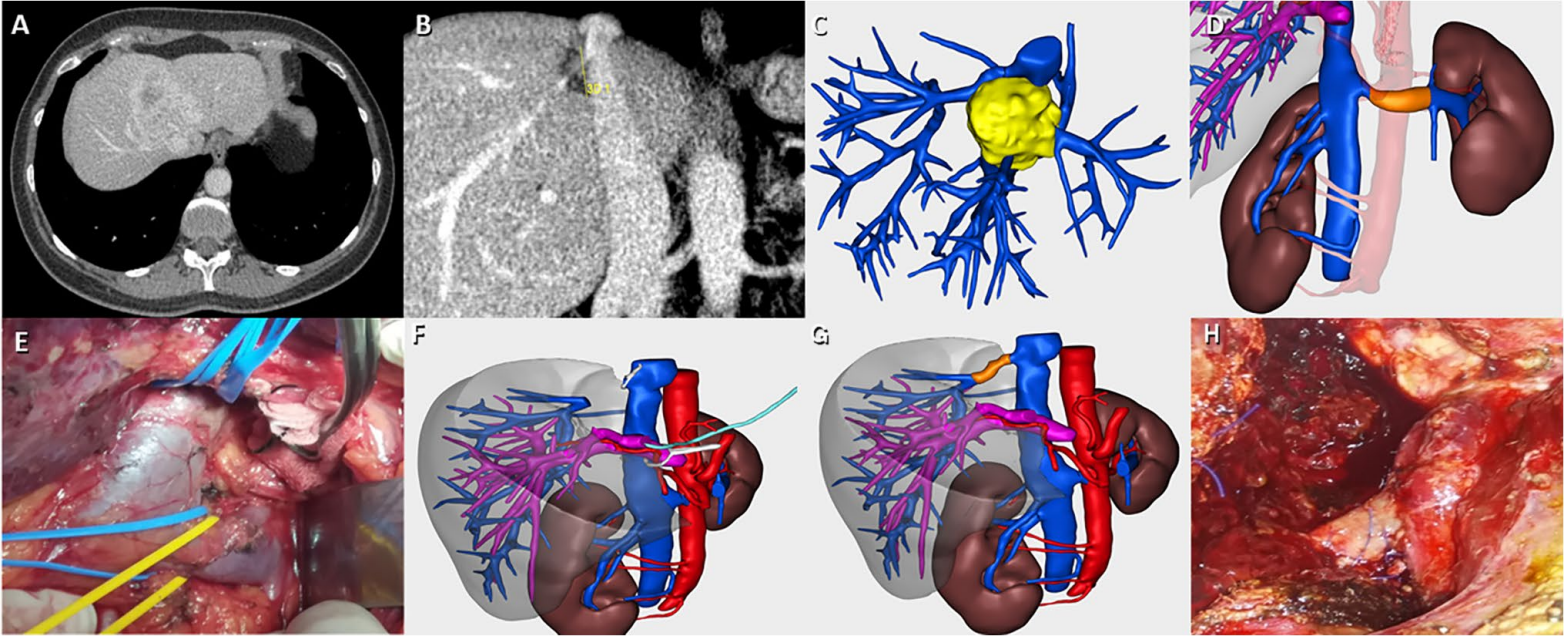
**A** Liver metastases with complete infiltration of the left, middle, and right hepatic vein. **B** Relation of the tumor lesion with the right hepatic vein and the vena cava. **C** Preoperative three-dimensional reconstruction of a liver metastasis at the confluence of the three hepatic veins. **D** Preoperative three-dimensional reconstruction of the future left renal vein graft (orange color). **E** Dissection of the left renal vein from its origin in the inferior vena cava to the gonadal vein which is respected to ensure an adequate drainage of the left kidney. **F** Preoperative three-dimensional reconstruction related to the left and middle hepatic vein control, right hepatic vein clamping, and left portal vein cannulation with the perfusion of hypothermic liver preservations. **G** and **H** Preoperative three-dimensional planning and final intraoperative final replacement of the hepatic vein repair with the left renal vein graft

**Fig. 2:**
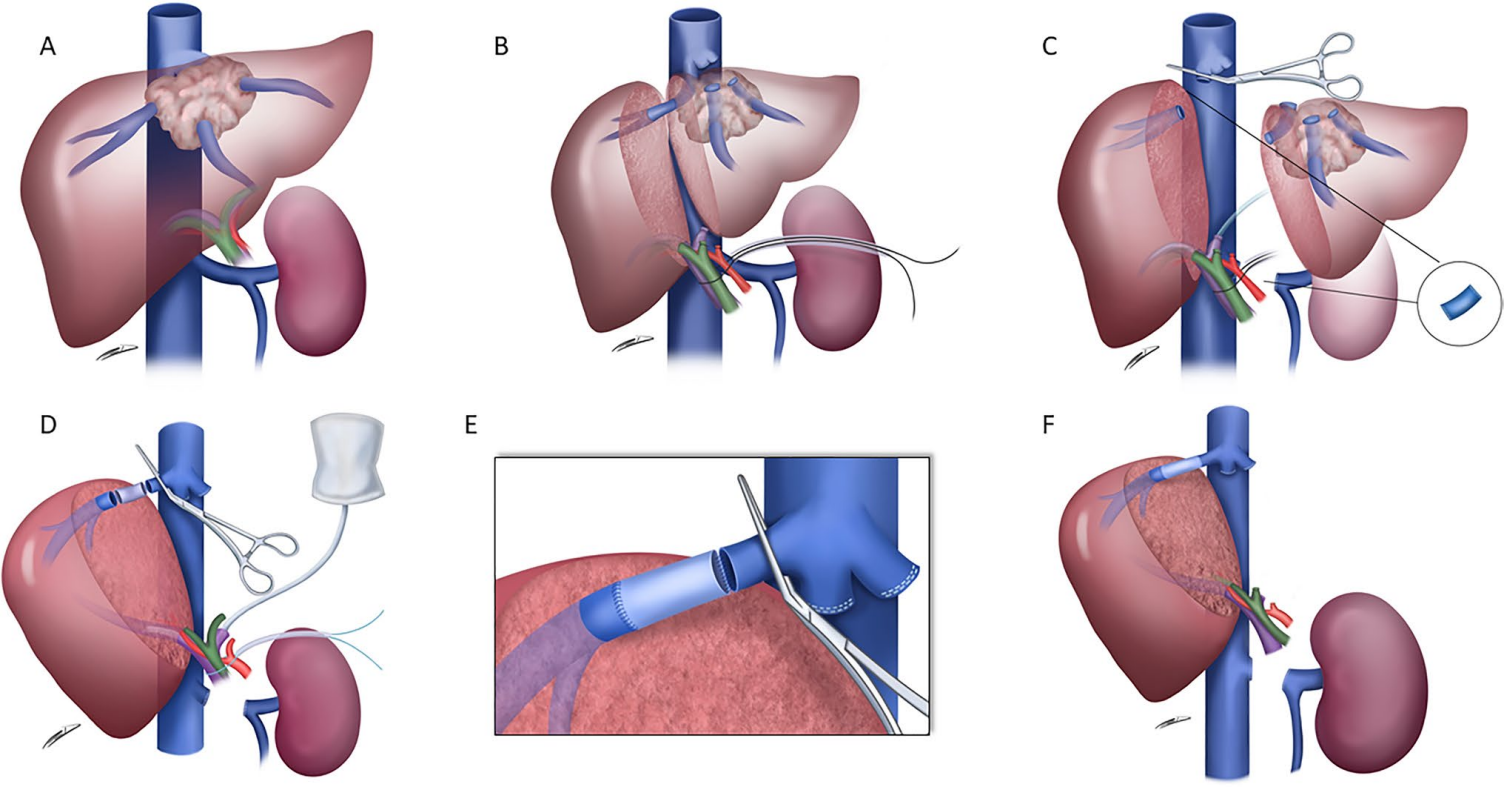
**A** Complete tumor invasion of hepatic veins. **B** Scheme about hepatic transection. **C** Left renal vein graft preparation and complete tumor resection. **D**-**E** Scheme on graft placement. **F** Final right hepatic vein reconstruction and hepatic reperfusion

Surgical technique was standardized and detailed in Figs. 1 and 2.

- Step 1 — staging and hepatic transection. The abdominal cavity was opened through subcostal bilateral incision and explored. Tumor location in relation to vascular structures was investigated with ultrasound. A band was placed around supra- and infrahepatic IVC and hepatic pedicle before the parenchyma transection. This was followed by dissection and ligation of the left hepatic artery along with dissection and ligation of a long pedicle of the left portal vein (for subsequent hepatic perfusion). Finally, a ligation and transection of the left bile duct was performed. Once the portal pedicle has been controlled, the hepatic partition with CUSA® or SonaStar® by Misonix is performed on the Cantlie line until the IVC is reached. The middle and left hepatic vein is divided and the section line is continued through segments 7–8 until reaching the proximity of the tumor that invades the right hepatic vein (RHV).
- Step 2 — LRV graft preparation. The LRV was isolated up to the gonadal vein. To obtain a longer LRV, the left adrenal vein was sacrificed. The IVC was clamped laterally to obtain a wide ostium of the LRV at the IVC level. Subsequently, the preparation of the graft was carried out, aiming to obtain a length of at least 4–6 cm.
- Step 3 — total hepatic vascular occlusion, tumor resection, and graft placement. For the left hepatectomy, a band was placed around the left and middle hepatic veins and a complete mobilization of the segment 1 was performed to the subsequent exclusion of the RHV. After completion of hepatic transection and left hepatectomy, the haptic pedicle was occluded with Pringle maneuver and RHV was clamped. This was followed by the cannulation of the left portal vein stump (at least 2 cm). The perfusion through left portal vein stump was started continuously with gravity and RHV was opened between tumor and IVC (Wisconsin solution, using 2–4 l at 4 °C). The preservation solution was hanged 80 cm above the patient. With this maneuver, it was prevented that the preservation solution could reach the central circulation. Flash fluid was removed with two aspirators to avoid a drop in body temperature. In addition, potassium level was controlled in regular intervals to monitor its level. Hepatectomy was completed by removing the tumor infiltrated part of the RHV. Once the tumor was resected, LRV anastomosis to the rest of RHV was performed first at the parenchymal level, subsequently at the proximal end of the IVC. The blood flow leaves through the RHV until the anastomosis of the RHV with the LRV graft is completed.
- Step 4 — hepatic reperfusion. Before finalizing the RHV reconstruction, albumin was infused via the left portal vein to remove potassium from the solution. Subsequently, RHV reconstruction was finalized, and left portal vein was ligated. Hepatic re-circulation was started first by opening RHV. At this point, the anastomosis was controlled via adequate hemostasis. Afterwards, the hepatic pedicle was opened as a final step of hepatic reperfusion. We checked that the venous graft had flow through ultrasound. Postoperatively, dose-adjusted subcutaneous heparin sodium (1 mg/kg/day for 1 month) was used, followed by warfarin and aspirin (5 and 75 to 100 mg/day for 3 months, respectively).

## Results

Four patients underwent left hepatectomy with vascular reconstruction using LRV for tumor invasion of the confluence of hepatic veins (Fig. S1). The details are shown in Table 1. The origin of the liver metastases was colorectal cancer (*n* = 3) and breast cancer (*n* = 1). All patients had received neoadjuvant chemotherapy. Median lesion size was 40 mm [interquartile range (IQR), 35–62].

**Table 1.** Demographic, perioperative, and oncological data of patients with a left renal vein graft for vascular reconstruction after resection of tumors in the confluence of the hepatic veins under vascular inflow occlusion with in situ hypothermic perfusion

N°	Diagnosis	Initial tumor stage	Gender	Age	Previous chemo	N° of lesions	Size (cm)	Blood losses (ml)	Surgical time (min)	Complications (Clavien)	Warm ischemia (min)	ICU length of stay (days)	Hospital stay (days)	Tumor recurrence	Survival time (months)
1	CRLM	T3N0M0	M	53	Xelox	1	40	1500	240	No	77	4	15	No	Alive (118)
2	CRLM	T3N1M1	F	60	Folfox + Cetuximab	2	40; 20	300	420	No	60	5	11	No	Death (90)
3	BCLM	T2N0M1	F	53	Anthracycline	1	70	500	300	Pleural effusion (IIIa)	62	7	15	Yes	Alive (98)
4	CRLM	T3N0M1	M	40	Folfiri + Cetuximab	1	34	500	480	Intraabdominal colección (IIIA)	72	5	24	No	Alive (6)

*CRLM*, colorectal liver metastases; *BCLM*, breast cancer liver metastases; *M*, male; *F*, female; *HTN*, hypertension; *CAD*, coronary artery disease; *rct*, resection; *RHV*, right hepatic vein; *MHV*, middle hepatic vein; *cm*, centimeters; *ml*, millimeters; *min*, minutes

### Hemodynamic parameters during surgery

**Fig. 3:**
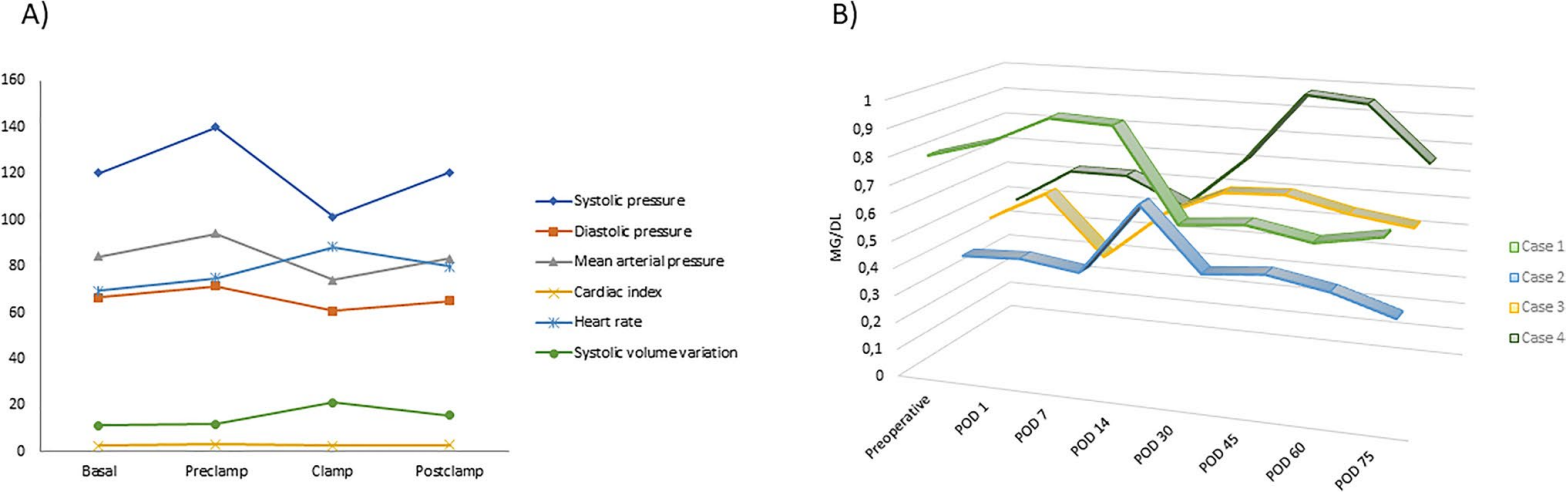
**A** Summary of hemodynamic alterations registered before, during, and after clamping during hepatectomy and graft placement. **B** Creatinine values in the immediate postoperative period and during follow-up

Systolic and diastolic blood pressure before, during, and after hepatic exclusion was 140 ± 4.58, 101.3 ± 16.5, 120.6 ± 13.2, 71.3 ± 5.6, 60.6 ± 7.37, and 65 ± 6 mmHg, respectively. Cardiac index was 3.2 ± 0.28, 2.7 ± 0.22, and 2.9 ± 0.2 and heart rate was 75 ± 12.8, 88.3 ± 6.5, and 80 ± 3.4 beats per minute (bpm), before, during, and after hepatic exclusion, respectively (Fig. 3A). End tidal CO_2_ and oxygen saturation values were stable with minimal variation during the surgery. Two patients required norepinephrine, the first of them prior and during hepatic exclusion. The second patient required prior, during, and after hepatic exclusion and dobutamine during and after hepatic exclusion. A third patient required dobutamine exclusively during hepatic exclusion. All but one patient required at least one packed red blood cell, one patient required a unit of pooled platelets, and two patients required 2 and 6 fibrinogen units.

### Surgical outcomes

Liver lesions infiltrated the RHV and the origin of middle and left hepatic veins in all cases. The median blood loss was 500 ml (IQR, 300–1500), median surgical time was 360 min (IQR, 255–465), and perfusion time with cold ischemia was 67 min (IQR, 60.5–77.5). The median length of the graft was 4.5 cm (IQR, 3.1–5.2). There were no major complications (≥ Clavien 3b) with a median hospital stay of 15 days (IQR, 12–21). Creatinine levels remained within normal range both in the immediate postoperative period and during follow-up (Fig. 3B). All patients could be resected with negative margin (R0). The grafts remained patent during follow-up without signs of thrombosis. Median follow-up was 97 months (IQR, 27–113). Three of four patients are currently alive. One patient had a lung recurrence that was treated with surgery (case 3). One patient died disease-free due to a cerebral hemorrhage (case 2).

## Discussion

This study suggests that hepatic veins resection within situ liver cooling and autologous LRV for hepatic vein reconstruction is feasible without increased morbidity and impairment of renal function. This situation is feasible only if there is no invasion of the cavo-hepatic junction because the control can perform by hanging of the left hepatic trunk and the anastomosis of the LRV is done on both sides of the RHV.

In healthy liver parenchyma, a liver remnant of around 25% is sufficient for safe liver resection [20–22]. Hepatic vein resection with reconstruction may be necessary if a tumor infiltrates to the hepatic veins. Such reconstruction is also proposed if the volume of the congestive area in liver remnant exceeds 20% [23]. Recent advances in liver machine perfusions may enable even ex vivo liver resection and vein reconstruction [24–27]. However, ex vivo liver resection may have the disadvantages of additional warm ischemia during portal and arterial anastomoses and is costly. An approach using in situ hypothermic perfusion is comparably simpler and more cost effective [28, 29]. Odhafer et al. [30] advocated a modified approach to ante situm resection, avoiding in situ hypoperfusion or veno-venous bypass during the ischemic phase of resection. Using this approach, they minimized ischemic time (mean 30.9 min) with a maximum duration of 65 min. However, the cooling with a preservation solution to reduce the risk of ischemia and reperfusion injury may be necessary if prolonged surgery is anticipated or the liver has underlying disease such as relevant macrosteatosis or chemotherapy-associated damage.

During clamping of the hepatic veins and IVC, the venous return may decrease up to 75%, which is hardly tolerable without adequate hemodynamic support. We decided on a supramaximal optimization protocol prior to the clamping test to compensate for the decline in venous return. First, a volume replacement is provided up to the upper limit of volume overload (determined by VVS < 10%), after which a higher volume administration does not achieve the goal of improving cardiac output [31, 32]. Following volume restoration, dobutamine infusion is administered to further increase the cardiac output while maintaining the heart rate below 120 bpm. In our experience, these measures have been sufficient for adequate hemodynamic management without requiring veno-venous bypass in any case.

For complex hepatic vein reconstruction, the experience has been obtained mainly from living donor liver transplantation [33–36]. Among the different possibilities of vascular grafts, the PTFE prosthesis has a higher risk of thrombosis, and a higher risk of infection via biliary leak, thus we propose to use PTFE prothesis as a last option. The use of cryopreserved veins can be an alternative for reconstruction, being an attractive option due to the variety of diameters, especially when a long length is required, but for this, a tissue bank must be available. It is important to select an autologous vein of optimal size and length, which might not always be available. In such case, an autologous vein could be also valuable alternative for reconstruction of hepatic veins. The external iliac vein has been used previously. However, resection of external iliac vein was associated with postoperative edema of the lower leg, as well as additional injuries. Nakamura et al. reported the use of external iliac vein grafts, superficial femoral vein, or a long saphenous vein graft for HV reconstructions, and only 3/8 grafts were viable at 30 months [37]. Other options such as the external jugular vein graft also require additional incisions in the neck [10]. The portion of the left renal vein is 5 to 6 cm long [38]. Different authors reported the possibility to use LRV grafts for vascular reconstruction in hepatobiliopancreatic surgery without a worsening of renal function in the short and long terms [13–18, 39–41] (Table S1). Indeed, it has several collateral branches (gonadal vein, the azygous-renal system, and splenorenal communications) that drain the venous return of the left kidney.

## Conclusions

The use of LRV for the reconstruction of the hepatic vein in tumors that infiltrate the hepatic veins is feasible without impairment of the long-term renal function. Correct hemodynamic management with adequate optimization of the patient prior to and during the clamping of the portal pedicle together with in cooling with preservation solution allows such extreme liver surgery and is alternative to ex vivo liver resection.

## Supplementary Information

Table S1 (DOCX 21 kb)

Fig. S1 (PNG 1466 kb)

High resolution image (TIF 399 kb)

## Data Availability

The authors confirm that the data supporting the findings of this study are available within the article and/or its supplementary materials.

## Code availability

The data for this project are confidential but may be obtained with Data Use Agreements with the Massachusetts Department of Elementary and Secondary Education (DESE). Researchers interested in access to the data may contact Ricardo Robles-Campos at rirocam@um.es. It can take some months to negotiate data use agreements and gain access to the data. The author will assist with any reasonable replication attempts for 2 years following publication.
