# Supplementary material for: Left renal vein graft and in situ hepatic perfusion in hepatectomy for complete tumor invasion of hepatic veins: hemodynamic optimization and surgical technique
Source: Langenbecks Arch Surg. 2022 Jan 31;407(4):1–7. doi: 10.1007/s00423-022-02451-6 (PMC9283147; doi:10.1007/s00423-022-02451-6)
Supplement: Supplementary file 1 — (DOCX 21 kb) [file 423_2022_2451_MOESM1_ESM.docx]

**Table S1.** Summary of the characteristics of the reviewed studies that describe the use of the renal vein graft to perform a vascular reconstruction in hepatobiliary malignances.

| **Author and year** | **N** | **Etiology** | **Age** | **Gender** | **Graft**  **Size** | **Surgical procedures** | **Operation time** | **Overall and graft complications** | **Creatinines** | **Survival outcomes** |
| --- | --- | --- | --- | --- | --- | --- | --- | --- | --- | --- |
| Miyazaki et al, 1995 | 3 | 2 CC  1 LM. | NA | NA | 4-5 | Tumoral+PV rct with LRV | NA | NA | Normal | Follow-up: 60m. |
| Miyazaki et al, 1997 | 6 | 1 CC.  3 LM.  2 GC | 66,5 (37-72) | 4M  2F | NA | Right trisegmentectomy + PV rct with LVR + hepaticojejunostomy  Caudate lobectomy + S2 wedge rst + PV rct with LVR  Extended right + PV rct with LVR + hepaticojejunostomy  PD+ hepatic resection of gallbladder bed+ PV rct with LVR  Right hepatectomy + S3 wedge resection+ right hemicolectomy+ IV rct: LVR graft.  Right hepatectomy with IVC rct with LVR | NA | 1 ileus.  1 liver failure + hepaticojejunostomy leak | Cr level (1 POD-14 POD): 0,76 / 0,7-3,31) | 1 died of liver failure (45POD)  1 died of recurrence (10m)  4 alive: 12, 9, 5 and 2,5 m |
| Suzuki et al, 2006 | 14 | 3CC  3LM  2 GC  5PADC  1 Mass-forming pancreatitis | 61,4 (37-76) | 7M  7F | NA | 1 Right hepatic trisectionectomy + bile duct  1 Caudate segmentectomy + S2 wedge  2 Extended right hepatectomy + bile duct  1 Right hepatectomy + S3 wedge rst + right hemicolectomy  2 Right hepatectomy  5 PD  1Pylorus-preserving PD + ioRT intraoperative radiation therapy  1 PD + cholecystectomy + gallbladder bed  10 Circumferential rst of the PV with LRV  1 lateral rst of the PV + patch repair with an LRV graft  3 lateral rst of the IVC with LRV patch rct | 9,19 (6,11-12) | 2 liver failure (died) | Preoperative Cr:  0,65 (0,36-0,91)  Postoperative Cr:  0,75 (0,43-3,31)  Follow-up Cr: 0,73±0,21 | Median survival was 16 m.  2 died of liver failure (28 and 45 d).  6 died of recurrence (42, 28, 22, 13, 10, 3 m.)  6 alive (67, 16, 7, 5, 4, 3m) |
| Smoot et al, 2007 | 9 | PADC | 57 | 7M  2F | NA | 3 standard PD and 6 pylorus-preserving PD  1 LVR patch graft for lateral edge of the SMV and PV  8 LVR interpostiton grafts: 1 PV, 1 between the SMV and PV with reimplantation of the SV. 1 between the SMV and PV without reimplantation of the SV. 5 were placed in the SMV, inferior to the confluence | 7,8 | 1 wound infection  1 delayed gastric emptying  1 GI bleeding  1 ascites and stenosis of the LRV interposition graft anastomosis (radiological treatment) | Preoperative: 0,93 (0,8-1,1)  Postoperative: 1,19 (0,8-1,5)  Follow-up (6,8m): normal | 2 had died 8,3 and 18,2 m of recurrent disease |
| Ohwada et al, 2007 | 11 | 1 LV.  1 HCC.  1 FNH.  5 PADC.  1CC  1 Schwannoma.  1 Colon carcinoma. | 54  (20-80) | 7M  4F | 3,64 (3-4) | 1 Extended left hepatectomy + S8 + rst MHV with LVR for V5-8 MHV  1 Segmentectomy (S7-8) + rst RHV with LVR for V5-6 RHV  1 Segmentectomy (S4, S7, S8) + rst RHV with LVR for V5-6-RHV  5 PD: 4 rct with LVR for PV, JV, IV and 1 PV-SMV  1 Extended right hepatectomy (+ S4a) + PD with LVR for PV-LPV  1 Right nephrectomy + para-renal IVC partial rst. with LVR graft +IVC patch + ovarian vein rct  1 Colectomy + cholecystectomy + duodenectomy + supra-renal IVC partial with LVR graft+ IVC patch + ovarian vein rct | NA | 1 stenosis (re-anastomosis)  2 PV thrombosis (1 thromboectomy and 1 re-anastomosis).  1 ascites  2 pleural effusion  2 delayed gastric emptying  1 splenic artery bleeding  1 liver failure (died) | Cr level after surgery was not increased except in the patient who died | 5 recurrences  1 died of liver failure (2 m)  5 died of recurrence (45, 20, 15, 13, 10 m)  5 alive (54, 51, 15, 11, 8 m) |
| Perumalla et al, 2008 | 1 | Alcholic liver cirrhosis | 48 | F | NA | Liver transplant: IMV was anastomosed to the LRV after ligating the distal IMV and anastomosed end to end with the donor PV. | NA | NA | Normal at 1 y | Alive at 1 y |
| Choudry et al, 2008 | 1 | PADC | 54 | M | NA | PD + SMV rct with LVR graft | NA | NA | Normal | NA |
| Choi et al,  2011 | 2 | PADC | 68  48 | 1M  1F | 3-3,5 | PD with rst of vein confluence en bloc with LRV | NA | 1 delayed gastric emptying. | Postoperative and follow-up CR between 0,33-1,16 | 1 died of recurrence (21m)  1 alive (23m). |
| Eguchi et al, 2019 | 1 | Intrahepatic CC | 79 | M | NA | Under extracorporeal circulation, left hepatectomy + IVC + RHV resection. RHV rct with reversed LRV and IVC graft | 18,6 h | Sepsis | NA | Died of sepsis (16 POD) |

AH: hepatic artery, CBD: common bile duct, CC: colangiocarcinoma, CRLM: colorectal liver metastases, FNH: focal nodular hiperplasia, GC: Gallbladder carcinoma, HA: Hepatic artery, HCC: hepatocellular cáncer, ICV: inferior cava vein, IMV: inferior mesenteric vein, ioRT: intraoperative radiation therapy, GI: gastrointestinal IV: ilecolic vein, JV: jejunal vein, LHA: Left hepatic artery, LM: liver metástasis, LVP: Left portal vein, LRV: left renal vein, NA: not aviable, PD: Pancreaticoduodenectomy, PADC: pancreatic adenocarcinoma, PV: portal vein, Rct: reconstruction, Itp: interposition, RGC: ringht gonadal vein, RII: right internal iliac vein, RHV: Rigth hepatic vein, MHV: Medial hepatic vein, POD: postoperative day, Rst: Resection, SMV: superior mesenteric vein, SV: splenic vein, UV: umbilical vein
