# Supplementary figures and images for: Left renal vein graft and in situ hepatic perfusion in hepatectomy for complete tumor invasion of hepatic veins: hemodynamic optimization and surgical technique
Source: Langenbecks Arch Surg. 2022 Jan 31;407(4):1–7. doi: 10.1007/s00423-022-02451-6 (PMC9283147; doi:10.1007/s00423-022-02451-6)

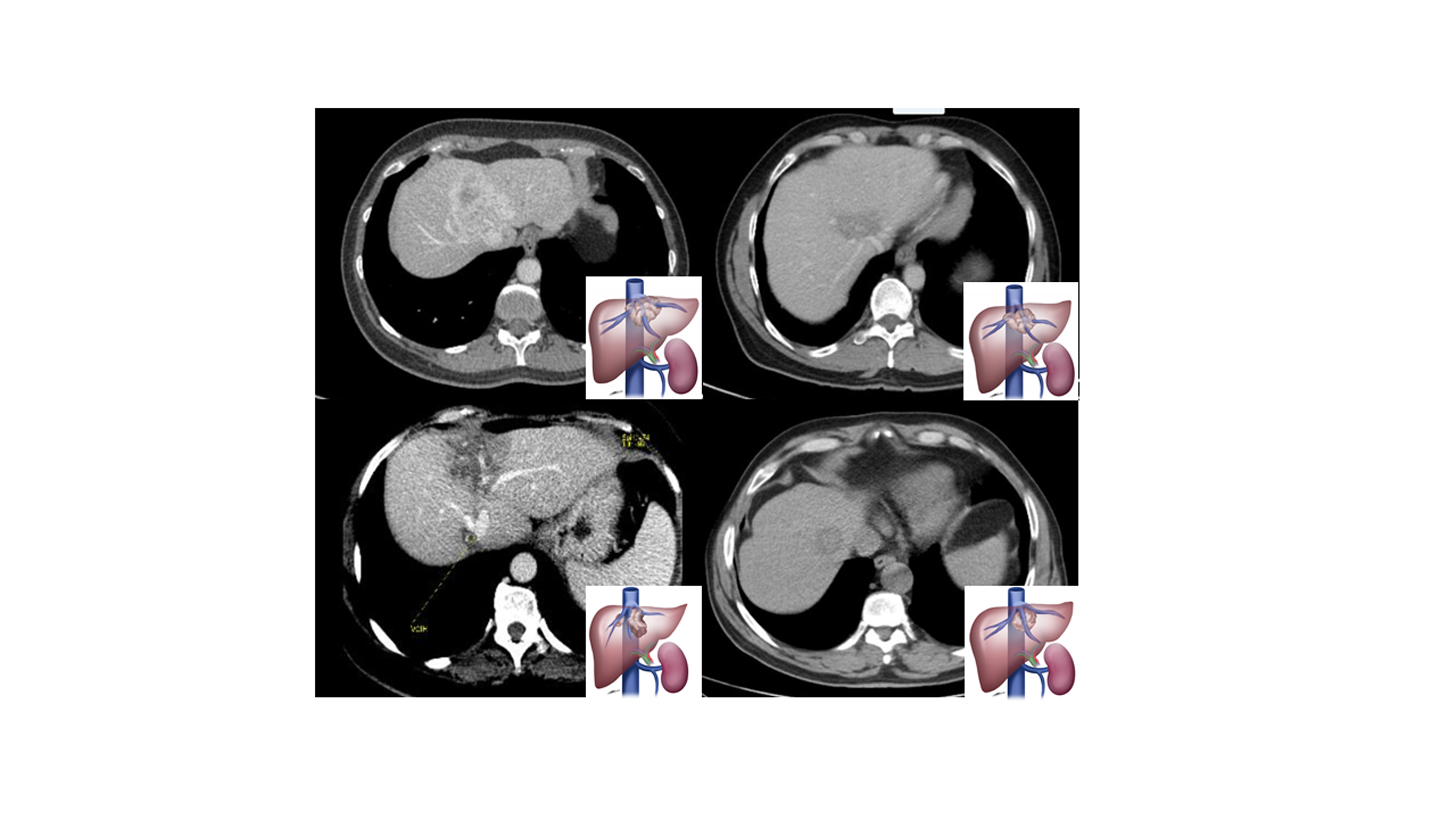

Supplement: Supplementary file 2 — (PNG 1466 kb) [file 423_2022_2451_Fig4_ESM.png]

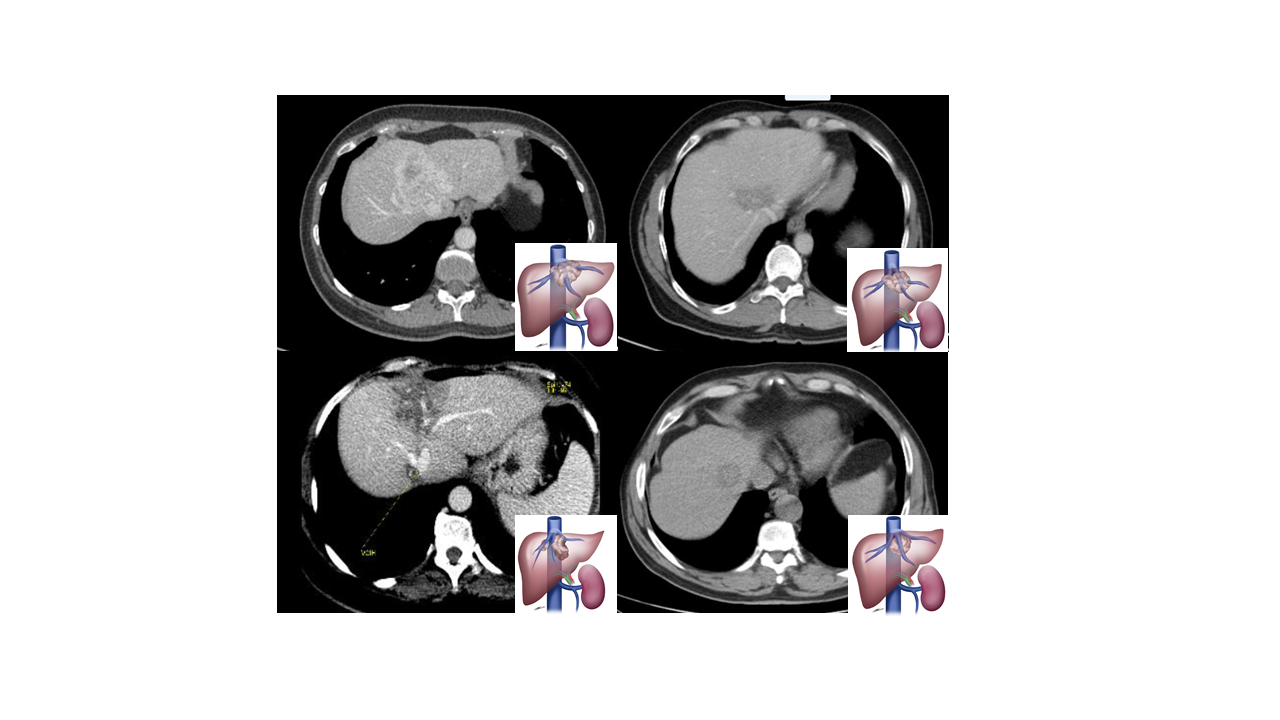

Supplement: Supplementary file 3 — High resolution image (TIF 399 kb) [file 423_2022_2451_MOESM2_ESM.tif]
